# Understanding Topological Insulators in Real Space

**DOI:** 10.3390/molecules26102965

**Published:** 2021-05-17

**Authors:** Angel Martín Pendás, Francisco Muñoz, Carlos Cardenas, Julia Contreras-García

**Affiliations:** 1 Departamento Química Física y Analítica, Universidad de Oviedo, 33006 Oviedo, Spain; ampendas@uniovi.es; 2 Departamento de Física, Facultad de Ciencias, Universidad de Chile, 9170124 Santiago, Chile; fvmunoz@gmail.com (F.M.); cacarden@gmail.com (C.C.); 3 Center for the Development of Nanoscience and Nanotechnology (CEDENNA), 9170124 Santiago, Chile; 4 Laboratoire de Chimie Théorique, Sorbonne Université and CNRS, 4 Pl Jussieu, 75005 Paris, France

**Keywords:** topological insulators, chemical bond, electron density

## Abstract

A real space understanding of the Su–Schrieffer–Heeger model of polyacetylene is introduced thanks to delocalization indices defined within the quantum theory of atoms in molecules. This approach enables to go beyond the analysis of electron localization usually enabled by topological insulator indices—such as IPR—enabling to differentiate between trivial and topological insulator phases. The approach is based on analyzing the electron delocalization between second neighbors, thus highlighting the relevance of the sublattices induced by chiral symmetry. Moreover, the second neighbor delocalization index, $\delta_{i,i+2}$, also enables to identify the presence of chirality and when it is broken by doping or by eliminating atom pairs (as in the case of odd number of atoms chains). Hints to identify bulk behavior thanks to $\delta_{1,3}$ are also provided. Overall, we present a very simple, orbital invariant visualization tool that should help the analysis of chirality (independently of the crystallinity of the system) as well as spreading the concepts of topological behavior thanks to its relationship with well-known chemical concepts.

## 1. Introduction

Topological insulators (TIs) constitute one of the big discoveries of solid state physics in the last decades [1,2,3,4,5]. TIs are materials with peculiar conducting properties: their bulk is insulating while localized conductive states are always present on their surface. These surface or edge states are topologically protected, meaning that this behavior is independent of the surface cleanness, disorder, passivation, etc.

These unique features have led to a growing interest in TIs in other areas of knowledge [6,7,8]. They have led to applications in quantum computing [9], low-power electronics [10], next-generation solar cells [11], molecular-based spintronics [12,13,14], photonics [15,16], novel organometalics [17], etc. As far as chemistry is concerned, the conducting surface states of TIs may be a game-changing in catalysis [18,19]. For instance, they could play the role of an electron reservoir that enhances the catalytic properties of noble metals supported on TIs [20,21,22] or even be used as modulators of crystallization [23].

However, predicting which systems will possess these interesting characteristics is not trivial. High-throughput electronic structure calculations have been carried out over complete databases of materials to find new TIs [24,25,26,27]. In addition, several topologically nontrivial materials have been computationally designed by means of large-scale calculations [28,29,30,31,32,33]. The methods usually applied to calculate the topological properties have been mainly developed for crystalline cases. However, in some situations, there is no such thing as a crystal lattice [34,35], and the bulk-boundary correspondence—a cornerstone in topological insulators—is not straightforward to follow. For these cases, a *local* probe of the symmetries giving rise to the topological order could be useful, especially one that provides chemical intuition.

Materials design usually passes through a conceptual understanding of the properties in real space, which is usually facilitated by the use of simple models [36,37,38]. With this aim in mind, we set up simple rules derived from quantum chemical topology that enable to identify trivial vs. topological behavior in a very simple model showing a trivial to topological transition, the Su–Schrieffer–Heeger (SSH) model of polyacetylene. This model, while being a textbook example, is still nowadays an intense subject of research in assembled molecules [39,40].

Section 2 is devoted to the theoretical background. Firstly, the fundamentals of the SSH model are given. We show that the bond-alternating polyacetylene model provides a good basis for understanding the meaning of edge states, topological protection and other basic concepts in the theory on TIs—all in terms of standard chemical concepts such as Lewis resonance structures and spatial (de)localization [41]. The concept of spatial delocalization is dwelled on in Section 2.2 by means of the Delocalization Index (DI) in the context of the quantum theory of atoms in molecules [42]. The evolution of DIs within the SSH model is analyzed in Section 3. We show how it enables identifying trivial, metallic and topological behavior in real space. By doing this, we are also able to visualize the differences between short and long polyacetyelene chains, i.e., when the turning point for the bulk behavior takes place. It should be noted that the descriptors that we derive are global descriptors of the wavefunction, invariant under orbital transformations and well defined at any level of theory, thus providing insights that are not easily accessible with other techniques employed in the theory of topological insulators. Finally, we show in Section 3.3 how the breaking of the chiral symmetry induced either by breaking the bipartite nature of the lattice or by destroying the equivalence of the two equivalent sublattices is reflected in the DI. This enables identifying the disappearance of the TI organization through DIs and to assign a simple bond order chemical meaning to their changes. The article finishes with a brief summary and main conclusions in Section 4.

## 2. Theoretical Background

### 2.1. The SSH Model

**Figure 1:**
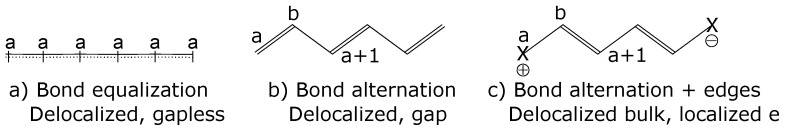
Chemical representation of the resonance forms of interest in 1,3,5-hexatriene.

Polyacetylene is the simplest conjugated polymer, with chemical formula (CH)${}_{2N}$. It is formed by alternating blocks of -CH- groups coupled by single and double bonds. Such simple model is very powerful, and it has been successfully used in the past to illustrate TI behavior. More specifically, Su, Schrieffer and Heeger made a very simple characterization of the wavefunctions of polyacetylenes by means of a tight-binding model [43]. Similar to the Hückel formalism, the hydrogen atoms in polyacetylene are ignored and only a single orbital, $\phi_{i}$, and a single electron per carbon atom is considered. To reproduce the bond alternation, two different first-neighbor hoppings, β and $\beta^{\prime }$, are necessary (see Figure 1b):

(1) $$\beta =-\langle \phi_{i,a}|\hat{H}|\phi_{i,b}\rangle ,\;\;\;\;\beta^{\prime }=-\langle \phi_{i,b}|\hat{H}|\phi_{i+1,a}\rangle ,$$

with the unit cell having two orbitals, $\phi_{i,a}$ and $\phi_{i,b}$. Note that the typical Hückel model is retrieved for bond equalization and $\phi_{i,a}=\phi_{i,b}$. The two non-equivalent sites of the unit cell imply the existence of two alternating sublattices of *a*- and *b*-labeled atoms.

**Figure 2:**
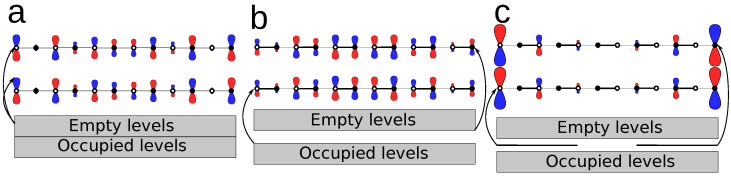
A scheme of the energy levels and HOMO/LUMO coefficients for: (**a**) the metallic (delocalized) case; (**b**) the trivial (main resonant form) case; and (**c**) the topological case (resonant form with edges).

In what follows, we set the on-site energy to zero (α=0) because all atoms are equivalent. A direct diagonalization of $\hat{H}$ without using periodic boundary conditions gives two qualitatively different outcomes, usually known in chemistry as two different resonance forms. The first one is known within the physics community as the *trivial* state, $\beta >\beta^{\prime }$; it corresponds to the resonance form with the biggest weight since it has no charges (Figure 1b). In this regime, there is an energy gap between the highest occupied (HOMO) and the lowest unoccupied (LUMO) molecular orbitals (Figure 2b).

The second solution, for which $\beta^{\prime }>\beta$, is a resonance form with two edge states (Figure 1c). Two new energy levels appear located in the middle of the band gap (i.e., at zero-energy, see Figure 2c); these become the new HOMO and LUMO orbitals of the system, which are non-bonding. From the representation of the orbitals in Figure 2c (bottom), we can see that, unlike the orbitals in the rest of the chain, the two non-bonding orbitals do not delocalize much over the bulk, but remain rather localized at the edges. Moreover, since they are formed from just two edges, a bonding and anti-bonding pair is formed (the phase opposition can be seen toward the right border). These pairs decouple as the chain length grows, becoming completely localized at the edges. This is equivalent to the typical result for the combination of two 1s H orbitals of H${}_{2}$ when they are taken infinitely away.

These edge-states are rather special: their existence is independent of the actual value of β and $\beta^{\prime }$ (as long as $\beta^{\prime }>\beta$). This fact explains the so-called “protection” of topological insulator properties upon changes in β and $\beta^{\prime }$, i.e., upon changes in the interaction strengths. The second solution of the SSH model can be understood as the simplest case of *topologically protected edge states*.

This leads to the bond-alternating polyacetylene having an extra symmetry known as “chiral symmetry”. Please note that the “chirality” concept is used as in condensed matter physics, not the common use of the “chiral” word in chemistry, associated to centers. Chiral symmetry sums up two characteristics: the polymer backbone (network) is entirely made of the same atoms and all the π electrons come from double bonds, so that the chain has 2N π electrons. These two features ensure that everything comes in pairs: all atoms have a “partner” (through the double bonds), and so do eigenvalues (energies) and eigenvectors (molecular orbitals). This explains the division into the sublattices a,b in Figure 1b,c. The fact that every eigenvalue of the Hamiltonian has a symmetric partner is the reason for the name of chiral symmetry. Physically speaking, we can define two sublattice projection operators, $P_{a},P_{b}$ such that $P_{a}+P_{b}=1$ and $P_{a}P_{b}=P_{b}P_{a}=0$. Chiral symmetry can be summarized as $H=P_{a}HP_{b}+P_{b}HP_{a}$, so that $P_{a}HP_{a}=P_{b}HP_{b}=0$. Although the model is simple, it is shown that it enables to understand the properties of real systems such as black phosphorous or graphene ribbons with known chemical concepts, and even to construct hand-waving arguments that would extend the model to higher dimensions and more robust (e.g., time reversal) symmetries [41].

The trivial and topological phases can be identified through the analysis of their eigenvalues and the shape of the HOMO and the LUMO. Indices, such as the IPR, have also been introduced that analyze localization of a given state. In all these cases, we need to identify a given one-particle state, e.g., an edge state. Moreover, in some cases, the crystal lattice might not be well defined, so that the bulk-boundary correspondence becomes fuzzy. For these cases, a *local* probe of the symmetries giving rise to the topological order could be useful.

In this paper, we aim to take a first step in this direction, characterizing the trivial and topological phases of the SSH model by means of delocalization indices. Moreover, since (de)localization indices refer to the full multielectronic wavefunction, these indices can be used when edge states are difficult to identify or isolate.

### 2.2. Localization and Delocalization in Real Space

A number of the structural features of the wavefunction of a chemical system may be unveiled by using chemical bonding descriptors. Among them, two- (or in general multi-) center bond orders are well known to the theoretical chemistry community, encoding information about the degree of electron delocalization among different centers [44]. In their most general real space formulation [45,46,47], they measure the multi-center fluctuation of electron populations, being only non-vanishing when the latter are mutually dependent. They can be obtained from domain-averaging the *n*th order electron cumulant densities [47] and are invariant under general orbital transformations. The simplest of all these descriptors is the two-center shared electron delocalization index (SEDI or simply DI [48]) which reduces in naïve cases to the chemical concept of bond order defined as the difference between the number of bonding and antibonding molecular orbitals of a molecule. Its general definition in real space uses the second order cumulant density, also known as exchange-correlation density,

(2) $$\rho_{xc}\left(\vec{1},\vec{2}\right)=\rho \left(\vec{1}\right)\rho \left(\vec{2}\right)-\rho_{2}\left(\vec{1},\vec{2}\right),$$

where ρ and $\rho_{2}$ stand for the standard spinless electron density and electron pair densities, normalized to *N* and N(N−1), respectively, *N* being the total number of electrons. All cumulants are size extensive, and $\rho_{xc}$ integrates to *N*. If we now partition space into regions *A* associated to atoms (or fragments) such that $⋃A=R^{3}$, then we induce a two-center partition of the number of electrons, $N=\sum_{A,B}N_{AB}$, where

(3) $$N_{AB}=\int_{A}d\vec{1}\int_{B}d\vec{2}\;\rho_{xc}\left(\vec{1},\vec{2}\right).$$

Since $\rho_{xc}$ is intimately linked to the Fermi–Coulomb hole, it is easy to show that $N_{AA}$ determines the number of localized electrons in region *A*, and that $N_{AB}+N_{BA}=\delta_{AB}$, the two-center delocalization index between centers *A* and *B* provides a measure of the delocalized population. Given the general non-local nature of $\rho_{xc}$, $\delta_{AB}=\mathrm{DI}\left(A,B\right)$ holds relevant information about the spatial distribution of electron correlations [49]. Notice that $N_{AA}$, which is also known in the literature as the localization index, LI(A) or $\lambda_{A}$, is a measure of the number of localized electrons in region *A*.

One of the ways to separate atoms A and B is through the quantum theory of atoms in molecules (QTAIM). This partitioning exhaustively divides the space using the topology induced by the gradient field of the electron density. This partition, proposed and developed by Bader and coworkers [42], has deep theoretical foundations, and is widely used. There are nevertheless many other proposals, both exhaustive and fuzzy [50]. For our purpose, a Hückel or tight binding approach in a lattice is equivalent to a condensation of the physical space into the nodes of the lattice. In this framework, tight binding orbitals labeled by a μ index, $\phi_{\mu }$, are expanded over the primitive functions at lattice sites *i* as $\phi_{\mu }=\sum_{i}c_{\mu }^{i}\chi^{i}$, and the DI between sites *i* and *j* may be trivially shown to be

(4) $$\delta_{i,j}=2{\left(\sum_{\mu }c_{\mu }^{i}c_{\mu }^{j}\right)}^{2},$$

**Figure 3:**
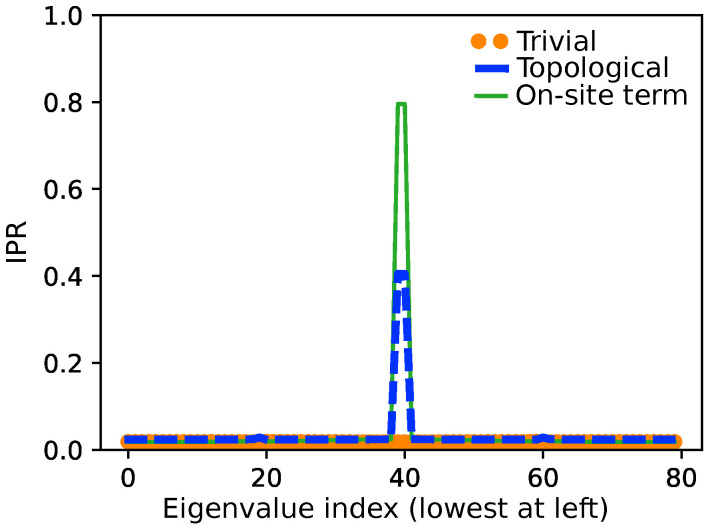
Inverse participation ratio (IPR) for each eigenvalue of a N = 80 SSH chain. The trivial ($\beta =3,\beta^{\prime }=1$), topological ($\beta =1,\beta^{\prime }=3$) and a non-topological case ($\beta =1,\beta^{\prime }=3,\Delta V=2$) were calculated. While the IPR is a an excellent tool to sort out edge states, it is not specific to the system’s chiral symmetry.

In the context of topological insulators, the degree of localization of one-electron states $\phi_{\mu }$ has also been measured by means of the so-called inverse participating ratio (IPR) [51], which in a tight-binding approximation is defined as IPR $=1//\sum_{i}{\left(c_{\mu }^{i}\right)}^{2}$. For a one-electron system, this is basically the inverse of $\delta_{i,i}=\lambda_{i}$. The latter quantity is related to the real space localization index [42], a measure of the variance of the population at a site. Notice that we need to identify a given one-particle state, e.g., an edge state, to define the IPR. Since delocalization or localization indices refer to the full multielectronic wavefunction, the indices used in our contribution are far more general, and they can be used when edge states are difficult to identify or isolate. It should also be noted that IPRs do not provide information on whether a state is topologically protected or not. Any trivial edge state may have an IPR similar to a topologically protected one (see Figure 3).

**Figure 4:**
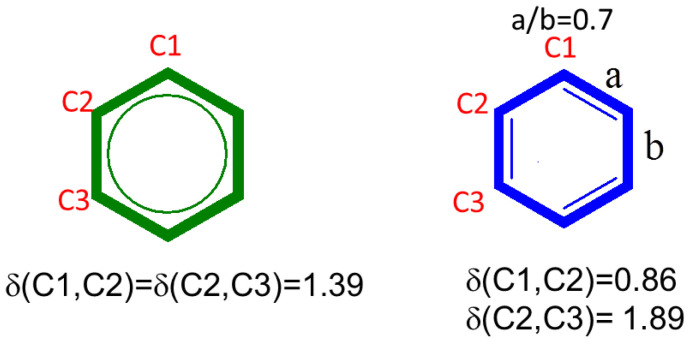
DI values for benzene and cyclohexatriene (with a/b = 0.7) calculated at the B3LYP/6-311G(d,p) level of theory.

Two-center DIs are used widely in high-level computational molecular chemistry. The nearest neighbor index, $\delta_{1,2}$, provides an orbital invariant descriptor of bond order. For instance, in a tight binding approximation, the C-C DI in ethane is exactly 1, and it is exactly 2 in ethylene. A delocalized cyclic form (to avoid borders) similar to that of Figure 4 (left) in cyclohexatriene (i.e., benzene) at the B3LYP/6-311G(d,p) level leads to a first-neighbor $\delta_{i,i+1}=1.4≃1.5$ for i∈1,N−1, whereas a localized one, as in Figure 4 (right) (a/b = 0.7), leads to $\delta_{a_{i}b_{i}}=1.9≃2$ and $\delta_{a_{i+1}b_{i}}=0.9≃1$.

DIs have also found their way to fill the language gap between the chemical and physical languages when applied to models. For instance, it has been shown [52] that DIs decay exponentially for insulators and in a power-law manner for metals, and this relation is analytical in the case of tight binding models. Similarly, DIs have been found to be related to Resta’s [53] localization tensor, so that their decay rate is rigorously related to the modern theory of polarization.

## 3. Real Space Characteristics of the SSH Model

### 3.1. Bond Alternation and Resonance

Given the ability of DIs to characterize metallic and insulating states in real space, the main aim of this paper is to characterize the SSH model, its trivial and topological phases, with the help of real space analysis techniques.

**Figure 5:**
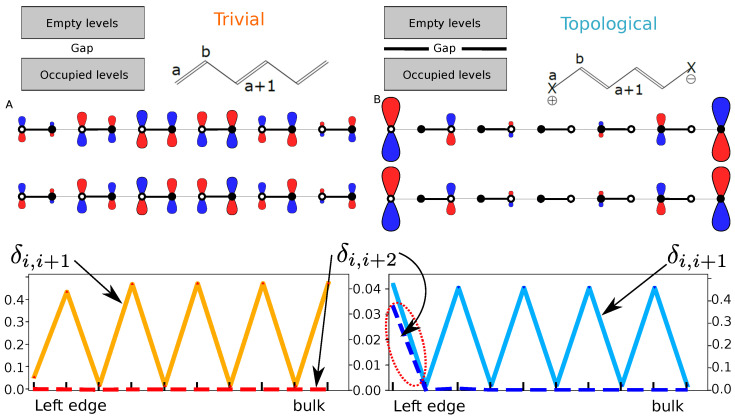
Energy levels, resonance scheme, orbitals around the Fermi level and DIs $\delta_{i,i+1}$ and $\delta_{i,i+2}$ of the bipartite lattice. The trivial phase (in red and orange, β=3 and $\beta^{\prime }=1$) and the topological phase (in blue and ligth blue, β=1 and $\beta^{\prime }=3$) are shown. The DIs have different scale, but the 0 coincides in all curves. Only 10 values of the DIs are shown, starting from an edge. The other edge is exactly symmetric to the one shown.

The first-neighbor, $\delta_{i,i+1}$, and second-neighbor, $\delta_{i,i+2}$, DIs for a chain of 80 atoms are shown in Figure 5. They represent the delocalization between the two sublattices ($a_{i},b_{i}$) and within the same one ($a_{i},a_{i+1}$), respectively.

The two phases (trivial and topological) lead to different bulk-like features in the $\delta_{i,i+1}$ (Figure 5). The position of the local maxima and minima of the first-neighbor $\delta_{i,i+1}$ for the two solutions are opposed. Recalling that $\delta_{i,i+1}$ gives an idea of bond order, this is related to the fact that the delocalized π electrons have changed position. Using the previous nomenclature into a,b sublattices, whereas the π bond is located in between $a_{i}$ and $b_{i}$ in the trivial phase, they are located in the complementary set ($b_{i}$ and $a_{i+1}$) in the topological one. Note that $\delta_{i,i+1}$ at the molecule borders points at the trivial edge states being more localized than those of the nontrivial case. In both situations, the localization of the edge quickly decays to its asymptotic bulk value.

We [41] previously showed that, within the SSH model, a charge situated on a given center will only delocalize along the same sublattice (be it $a_{i}$ or $b_{i}$, i=1,N). This result is well-known in chemistry: ortho charges in a benzene ring only delocalize in ortho and para positions, but not in meta. This is a result of chiral symmetry, which forces all non-zero energy states to be equally supported, i.e., to have equal overall coefficients, on the two sublattices. On the contrary, the zero-energy states present in the gap of topological phases can be chosen to have coefficients from only one of the sublattices (e.g., a symmetric and antisymmetric mix of orbitals in Figure 5).

Hence, the next nearest neighbor delocalization should distinguish trivial from topological systems. This can be easily demonstrated by looking at the second-neighbor delocalization index i.e., $\delta_{i,i+2}$, in Figure 5 (the relevant result is highlighted by the red dashed circle). Whenever chiral symmetry exists and non-zero energy states arise, the $\delta_{i,i+2}$ index should vanish. By taking into account Equation (4), it is found that only the zero-energy states will contribute to $\delta_{i,i+2}$, and as these edge state delocalizations, $\delta_{i,i+2}$, will differ from zero only close to the edges. This means that for sufficiently long chains, the bulk-like region of the bipartite lattice $\delta_{i,i+2}$ is zero. Moreover, this happens regardless of the phase: trivial, topological or metallic. Chemically, this reflects the fact that if a charge is situated on a given center $b_{i}$, resonance forms will only delocalize the charges along the $b_{i}$ (i=1,N) centers. Of course, the same applies to the *a* sublattice. For a long chain such as the one we are looking at, the edge states are effectively decoupled and $\delta_{1,i+2}\neq 0$ only for edge states, e.g., $\delta_{1,3}$ (Figure 5, right).

**Figure 6:**
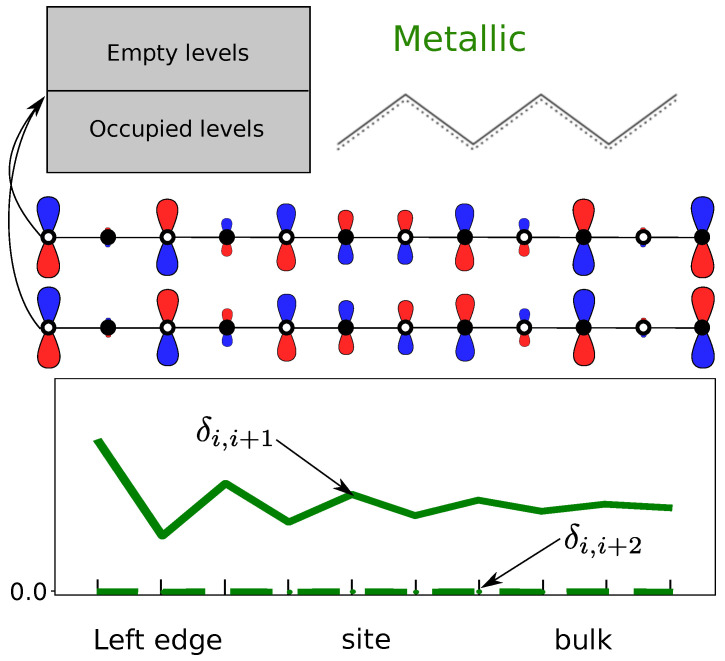
Energy levels, resonance scheme, orbitals around the Fermi level and DIs $\delta_{i,i+1}$ and $\delta_{i,i+2}$ of the bipartite lattice in the metallic case. The chain is 80 atoms (only 10 sites are shown) and $\beta =\beta^{\prime }=2$.

It is also interesting to analyze $\delta_{i,i+1}$ in the intermediate metallic case ($\beta =\beta^{\prime }$). Figure 6 shows how the metallic behaviour differs from the localized case. Whereas $\delta_{i,i+1}$ decays exponentially in the non-metallic states ($\beta \neq \beta^{\prime }$), the metallic state shows the typical polynomial decay [54]. This approach makes it possible to identify from the wavefunction the localization schemes. While the π electrons are delocalized for $\beta =\beta^{\prime }$, they are localized for $\beta \neq \beta^{\prime }$. From the computational point of view, localization schemes (e.g., Foster–Boys) would lead to a unique answer in the $\beta \neq \beta^{\prime }$ case. Instead, the localization for $\beta =\beta^{\prime }$ would not be unique.

Hence, the simultaneous analysis of delocalization patterns, $\delta_{i,i+1}$ and $\delta_{i,i+2}$, enables us to identify a chiral setup along with the different phases. While $\delta_{i,i+1}$ provides insight on the existence of resonance, $\delta_{i,i+2}$ confirms the existence of chirality and the existence of two distinct phases, one of them with two edges, i.e., the topological phase.

### 3.2. Short vs. Long Chains: Simulating Crystalline Environments

It should be noted that crystallinity is needed to build a topological insulator model that effectively decouples the edges. This makes it very tricky to choose the correct chain length where edges are effectively decoupled. Rigorously speaking, this can be achieved by having just one border (semi-infinite boundary conditions), which is hard to implement. This question is often overlooked and one relies on intuition or common sense in order to choose the correct chain sizes. However, the task becomes easier when looking at the delocalization indices.

**Figure 7:**
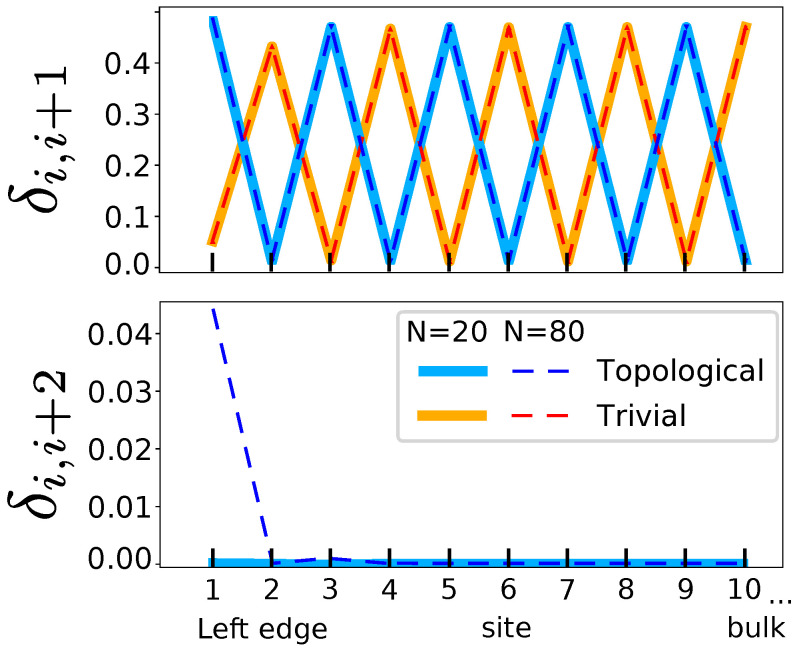
DIs $\delta_{i,i+1}$ and $\delta_{i,i+2}$ of the bipartite lattice with 20 and 80 atoms (only 10 sites are shown). The index $\delta_{i,i+2}$ for trivial insulators is zero everywhere.

We represent the delocalization index for a short (20 atoms) and a long chain (80 atoms) SSH model in Figure 7. Larger chains show behaviors indistinguishable from the N=80 case. In the figure, the curves of $\delta_{i,i+1}$ are practically identical. However, the result drastically differs for $\delta_{i,i+2}$. We can see that $\delta_{1,3}\approx 0$ for the topological phase of the small chain—a consequence of a small but non-negligible interaction between the edges.

This can be used to try to establish a quantitative transition length between uncoupled and coupled edges. By looking at the $\delta_{i,i+2}$, we find that the crossover from short to long chains happens at N∼70 atoms, which is roughly 10 nm [55]. To provide some real-life comparison, experiments with 3D topological insulators show that crossovers from coupled to decoupled surface states occur at a slab thickness of ca. 6 nm [56]. Hence, the SSH model coupled to the DI calculation provide a reasonable measure of the order of magnitude needed to build a topological insulator.

### 3.3. Revealing the Breaking of Chiral Symmetry

In this section, we show that second neighbor bond orders enable the effective identification (and quantification) of the presence/absence of chiral symmetry.

#### 3.3.1. The Number of Centers

**Figure 8:**
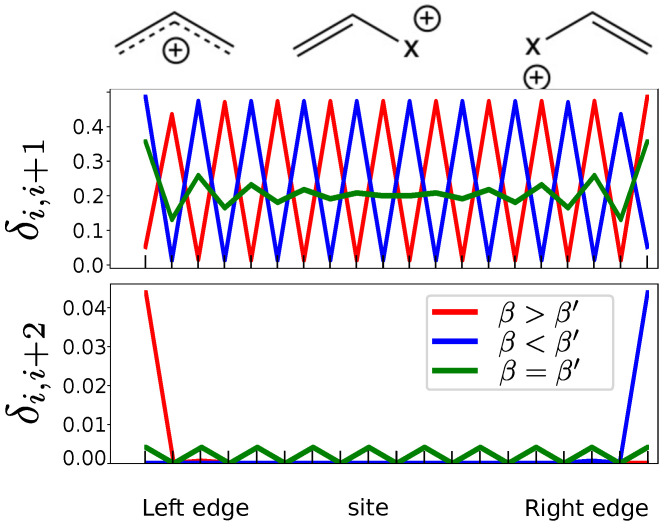
Resonance schemes, $\delta_{i,i+1}$ and $\delta_{i,i+2}$ for a chain with N=21 atoms.

We explain above in the theoretical background that chiral symmetry requires 2*N* centers. Let us see what happens to the delocalization index when the chain has an odd number of centers. For *N* = 21 atoms, the delocalization index $\delta_{1,3}$ (see Figure 8) is non-zero only at one edge—the position of the non-bonding atom. Increasing β to a value larger than $\beta^{\prime }$ just changes the position of the non-bonding atom to the other edge. The index $\delta_{i,i+1}$ at one edge of the molecule behaves in the same way as in the trivial phase (Figure 5), and as the topological phase at the other edge. This reflects the absence of two differentiated phases, and hence the absence of chirality. The corresponding equivalent resonance forms are shown on the top of Figure 8.

#### 3.3.2. The On-Site Energy

**Figure 9:**
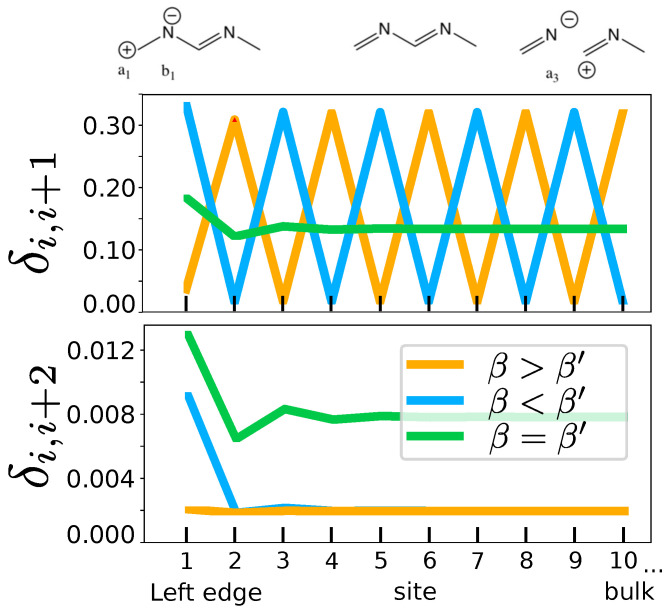
Resonance schemes, $\delta_{i,i+1}$ and $\delta_{i,i+2}$ for the di-atomic lattice with an on-site term (ΔV=−2) added to the second sublattice. The chain is 20 atoms long (only 10 sites are shown) and the hoppings $\beta ,\beta^{\prime }$ are the same as in Figure 5.

The other condition for having a bipartite lattice is that all atoms be equal. If a different on-site potential is introduced on one of the sublattices, i.e., $\Delta V=\alpha_{a}-\alpha_{b}\neq 0$, chirality is also lost. Chemically, this is equivalent to introducing impurities (Figure 9, top, shows the example with nitrogen atoms).

Once again, the shape of the $\delta_{i,i+1}$ index is qualitatively unaffected by the breaking of the chiral symmetry (Figure 9). However, the loss of chirality can be easily verified by returning to $\delta_{i,i+2}$. Instead of the zero value obtained when chiral symmetry is present, $\delta_{i,i+2}$ is finite everywhere in the bulk. This provides with an easy a visual characterization of doping in a chiral symmetry induced topological insulator.

## 4. Conclusions

Chirality in the Su–Schrieffer–Heeger model is related to the existence of a bipartite lattice, and hence to pair-wise energies. This chiral symmetry leads to the existence of two phases, trivial and topological insulator. Within the latter, two of the paired energies appear at zero energy. When we go from the topological insulator to the trivial phase, these zero energy states shift. However, the chirality is still present. This transition can be identified by basic bonding descriptors, such as covalent bond orders (delocalization indices). More specifically:

- The chiral symmetry is present if bond orders with second-neighbors, $\delta_{i,i+2}$, are zero in the bulk (i.e., equivalent to the absence of delocalization in meta carbons in benzene).
- A topological transition in the bulk can be detected by a change in the pattern of maxima and minima bond orders with first-neighbors, $\delta_{i,i+1}$, which reflects the two resonance forms.
- The topological phase and its protected edge states can be detected by a non-zero second-neighbor bond order at both edges of the molecule. These bond orders decay exponentially to zero far from the edges.
- The visualization of these edge states enables the quantification of edge decoupling, i.e., of the appearance of bulk properties in linear chains.

Moreover, the loss of chirality itself is related to the absence of a bipartite lattice, as well as identified by the $\delta_{i,i+2}$ pattern:

- In the case of impurities, $\delta_{i,i+2}\neq 0$ due to the on-site ΔV.
- In the case of odd number of atoms, only one edge state appears upon changing from $\beta >\beta^{\prime }$ to $\beta <\beta^{\prime }$, showing that both cases are equivalent, so that there is no phase transition.

Finally, it should be noted that delocalization indices show two advantages with respect to other commonly used localization methods, such as the inverse participation ratio (IPR). On the one hand, since DIs are fully orbital invariant objects that characterize the full wavefunction of a system and can be obtained at any level of theory, we expect that their study not only in model systems but in actual materials will lead to new insights. Moreover, since suitable generalizations to many centers exist, there is in principle no restriction to the dimensionality of the system explored. Work in this direction is currently in progress. On the other hand, IPRs do not give hints about whether a state is topologically protected or not. Any trivial edge state may have an IPR similar to a topologically protected one. DIs provide a local measure of the chiral symmetry of the system and states, thus—at least for the case of chiral symmetry—the DIs works as a probe of the symmetries that allows the topological classification. 

## Data Availability

All data are available from the authors upon request.

## Sample Availability

Samples of the compounds are available from the authors.
